# Growth, body composition, and cardiovascular and nutritional risk of 5- to 10-y-old children consuming vegetarian, vegan, or omnivore diets

**DOI:** 10.1093/ajcn/nqaa445

**Published:** 2021-03-19

**Authors:** Małgorzata A Desmond, Jakub G Sobiecki, Maciej Jaworski, Paweł Płudowski, Jolanta Antoniewicz, Meghan K Shirley, Simon Eaton, Janusz Książyk, Mario Cortina-Borja, Bianca De Stavola, Mary Fewtrell, Jonathan C K Wells

**Affiliations:** Childhood Nutrition Research Centre, UCL Great Ormond Street Institute of Child Health, University College London, London, UK; Department of Pediatrics, Nutrition, and Metabolic Diseases, The Children's Memorial Health Institute, Warsaw, Poland; Department of Pediatrics, Nutrition, and Metabolic Diseases, The Children's Memorial Health Institute, Warsaw, Poland; MRC Epidemiology Unit, University of Cambridge School of Clinical Medicine, Cambridge, UK; Department of Biochemistry, Radioimmunology, and Experimental Medicine, The Children's Memorial Health Institute, Warsaw, Poland; Department of Biochemistry, Radioimmunology, and Experimental Medicine, The Children's Memorial Health Institute, Warsaw, Poland; Department of Nephrology, Kidney Transplantation, & Hypertension, The Children's Memorial Health Institute, Warsaw, Poland; Department of Nutrition, School of Public Health, University of São Paulo, São Paulo, Brazil; Developmental Biology and Cancer Research and Teaching Department, UCL Great Ormond Street Institute of Child Health, University College London, London, UK; Department of Pediatrics, Nutrition, and Metabolic Diseases, The Children's Memorial Health Institute, Warsaw, Poland; Population, Policy, and Practice Research and Teaching Department, UCL Great Ormond Street Institute of Child Health, University College London, London, UK; Population, Policy, and Practice Research and Teaching Department, UCL Great Ormond Street Institute of Child Health, University College London, London, UK; Childhood Nutrition Research Centre, UCL Great Ormond Street Institute of Child Health, University College London, London, UK; Childhood Nutrition Research Centre, UCL Great Ormond Street Institute of Child Health, University College London, London, UK

**Keywords:** stature, bone mineral content, iron deficiency, vitamin B-12 deficiency, vitamin D deficiency, cardiovascular risk, vegetarian children, vegan children

## Abstract

**Background:**

Plant-based diets (PBDs) are increasingly recommended for human and planetary health. However, comprehensive evidence on the health effects of PBDs in children remains incomplete, particularly in vegans.

**Objectives:**

To quantify differences in body composition, cardiovascular risk, and micronutrient status of vegetarian and vegan children relative to omnivores and to estimate prevalence of abnormal micronutrient and cholesterol status in each group.

**Methods:**

In a cross-sectional study, Polish children aged 5–10 y (63 vegetarian, 52 vegan, 72 matched omnivores) were assessed using anthropometry, deuterium dilution, DXA, and carotid ultrasound. Fasting blood samples, dietary intake, and accelerometry data were collected.

**Results:**

All results are reported relative to omnivores. Vegetarians had lower gluteofemoral adiposity but similar total fat and lean mass. Vegans had lower fat indices in all regions but similar lean mass. Both groups had lower bone mineral content (BMC). The difference for vegetarians attenuated after accounting for body size but remained in vegans (total body minus the head: –3.7%; 95% CI: –7.0, –0.4; lumbar spine: –5.6%; 95% CI: –10.6, –0.5). Vegetarians had lower total cholesterol, HDL, and serum B-12 and 25-hydroxyvitamin D [25(OH)D] without supplementation but higher glucose, VLDL, and triglycerides. Vegans were shorter and had lower total LDL (–24 mg/dL; 95% CI: –35.2, –12.9) and HDL (–12.2 mg/dL; 95% CI: –17.3, –7.1), high-sensitivity C-reactive protein, iron status, and serum B-12 (–217.6 pmol/L; 95% CI: –305.7, –129.5) and 25(OH)D without supplementation but higher homocysteine and mean corpuscular volume. Vitamin B-12 deficiency, iron-deficiency anemia, low ferritin, and low HDL were more prevalent in vegans, who also had the lowest prevalence of high LDL. Supplementation resolved low B-12 and 25(OH)D concentrations.

**Conclusions:**

Vegan diets were associated with a healthier cardiovascular risk profile but also with increased risk of nutritional deficiencies and lower BMC and height. Vegetarians showed less pronounced nutritional deficiencies but, unexpectedly, a less favorable cardiometabolic risk profile. Further research may help maximize the benefits of PBDs in children.

## Introduction

Recently, interest in plant-based diets (PBDs) has increased in many global regions. Although formal estimates are lacking, numerous sources indicate that more people are adopting meat-free diets in industrialized countries (1, 2). Broadly, vegetarian diets exclude meat and fish, whereas vegan diets eliminate all products of animal origin, including dairy and eggs (3). There are 3 main reasons for their rising popularity: planetary sustainability; improving health, including prevention of noncommunicable disease (NCD); and heightened concern for animal welfare (4, 5). The first two have been recently reflected in healthy eating recommendations by numerous international health organizations (5, 6). These issues primarily concern adults, who may then act on them when selecting diets for their offspring. The health effects of vegetarianism and veganism have been evaluated in adults and include lower cardiometabolic risk (7) but increased fracture risk in vegans with low dietary calcium content (8).

Less evidence is available for children. Atherosclerosis originates in childhood and relates to cardiometabolic risk factors that, along with dietary habits, track into adulthood. Therefore, PBDs in childhood might reduce adult risk of cardiovascular disease (CVD) (9); however, any such benefits must be considered in light of safety in the pediatric population. Vegetarians and vegans restrict intake of whole food groups. This is of particular concern in children, whose nutrient and energy needs are higher relative to body weight and whose growth might be impaired by nutrient deficiencies at sensitive periods of development (10). Existing data come from studies of heterogeneous design and relate predominantly to anthropometric outcomes and to vegetarian children. Previous work on vegetarian children showed normal growth and a tendency to be leaner compared with omnivores (11). Evidence on blood micronutrient status for this group is available primarily for iron status, showing wide variation in the prevalence of deficiency (12). Data on other blood parameters are scant (11, 13). There are no current informative studies on vegan children other than those <3 y old (14) when health effects might be less evident.

The sparsity of evidence contributes to inconsistencies between medical and nutrition organizations’ statements regarding the safety of meat-free diets in childhood (15–19). Given growing global campaigns to encourage PBDs, reliable evidence is urgently needed, so that these diets can help decrease ecological damage while also promoting health in both adults and children. We aimed to evaluate differences in several indicators of health, including growth, body composition, CVD risk, and micronutrient status, along with estimating the prevalence of inadequate serum micronutrient and abnormal cholesterol status in vegetarian or vegan children, relative to an omnivore reference group.

## Methods

### Study design

A cross-sectional methodology was chosen for this study. Although intervention trials are ideal for providing evidence for a causal relation, it is unethical and unfeasible to randomly allocate healthy children to different dietary regimens of unknown health effects for periods long enough to elicit effects on growth, body composition, or selected CVD risk factors. Although our study is cross-sectional, the exposure tracks back into the past (i.e., the children recruited to the study had to have followed their respective diets for at least 1 y, and their diet was measured within 2 wk before the outcome data collection took place).

### Subjects

We studied healthy Polish children (aged 5–10 y), all of white European ethnicity. All children had to have followed their diet for ≥1 y prior to participation. Exclusion criteria included receiving any treatment other than bronchodilators and/or steroids for asthma or conditions adversely affecting growth and development. The latter included obesity and wasting, defined using age-specific pediatric international BMI (in kg/m^2^) cutoffs, corresponding to 30 at age 18 y and –2 *z* scores respectively (20, 21), as these suggest malnutrition regardless of dietary choice, and height <5th percentile for Polish growth curves (22) due to a diagnosed growth disorder. Eligibility was established via electronic questionnaires sent to parents before the study and confirmed during data collection.

### Recruitment and sampling

Vegan and vegetarian children were recruited by advertisements using Internet portals and social media, targeting issues of vegetarianism and veganism. Omnivores were recruited by asking vegan and vegetarian children to bring a friend of the same sex and similar age (within ±1-y difference). In addition, advertisements were placed in health food stores and on Internet portals devoted to healthy eating, from which omnivores were matched to vegetarians and vegans by sex, age (±1 y), maternal education (higher, secondary, primary), and place of residence (urban compared with rural).

The sample size per group was calculated using data for blood lipids (total and LDL cholesterol) from a pilot study, investigating blood lipid concentrations in healthy Polish prepubertal children on vegan (*n* = 46) or vegetarian (*n* = 29) diets in comparison with age- and sex-matched omnivores (*n* = 61) in 2010. We aimed to detect, with 80% power and a significance level α of 0.05, mean differences ≥0.5 *z* score between omnivore and either vegan or vegetarian groups in each outcome, requiring 64 children per group. Anticipating occasional missing data, we intended to recruit 66 children per group. We specified age groups for recruitment, taking into account both the scarcity of vegan children in Poland and the aim of achieving similar age distributions across dietary groups. We aimed to recruit 7 of each sex-diet combination at 5 y and 13 in the 6- to 7-year and 8- to 10-year age groups (total 198). Recruitment lasted from June 2014 until July 2016.

### Background characteristics

The following family data were collected before enrollment via an electronic questionnaire: child's date of birth, parent-reported weight and height, current health status, medications, information on parental smoking and educational attainment, crude information on income level per person in the household, family size, family history of NCD (parental/grandparental hypertension, obesity, diabetes, or coronary artery disease or myocardial infarction before age 55 y for men and 65 for women), religion, breastfeeding/formula feeding practices, and the present and past frequency of animal product consumption. During recruitment, additional questionnaires in the clinic ascertained the child's birth order, fracture history, lactose intolerance status, birth weight, Apgar score, gestational age, self-reported parental height, maternal prepregnancy nutritional status (weight, nutritional supplementation practices, dietary practices), and if the child had been on holiday with significant sun exposure recently.

### Physical activity

Physical activity (PA) was measured by Actigraph GT1M accelerometers. Children were asked to wear an accelerometer on the right hip during waking hours for 4 d. A minimum of 2 d with ≥8 h of activity recordings was deemed valid (23, 24). We used average counts per minute (CPM) as an indicator of overall activity. In addition, time spent in sedentary, moderate, and vigorous PA was extracted to compare time spent at different PA intensity levels between dietary groups.

### Exposure—dietary assessment and categorization

Prior to recruitment, parents completed a screener questionnaire to quantify the child's frequency of consumption of meat, fish, dairy products, and eggs in the past 12 mo. The screener questionnaire was used to recruit and classify children as omnivore, vegetarian, or vegan and to assess the frequency of animal product consumption from birth.

Food diaries were used to assess dietary intake. Parents/guardians recorded everything eaten or drunk over 4 consecutive days, including 2 weekend days. The records were obtained within the 2 wk before physiologic data collection, as most of the blood biochemicals of interest respond to dietary changes within that time (25–27). Thorough written instructions, along with pictures of household measures of food and drinks, were provided. Two telephone calls were made to explain the written instructions, to answer questions, and to check compliance. Involvement of school or kindergarten staff in keeping the record prospectively was encouraged. If insufficient details were obtained by parents on the composition of meals eaten outside of the home, schools, kindergartens, or restaurants were directly contacted by the research team. The staff provided recipes of meals cooked or served and information on the quantity of foods consumed by children at their eating establishment.

Estimated food intakes were entered into nutritional analysis software (Esha Food Processor, version 10.14) by 2 dietitians. Polish food composition tables (28) linked to the software were used as the primary reference for calculating nutrient intakes. Nutrient content of foods not available in the Polish tables (e.g., vegetarian-specific foods) was obtained from the database of the USDA (29). Final classification into dietary groups was performed after analyzing the food diaries. Participants were classified as vegan if they consumed no flesh foods (meat and fish) or other animal-based products (eggs, dairy) for at least the previous year or if they consumed no flesh foods (meat and fish) and nearly no other animal-based products (eggs, dairy) over the past year, with minor exceptions that amounted to <5% of dietary energy from eggs and dairy estimated from the food diary. The dietitians responsible for diary data entry were blinded to this cutoff value. Vegetarians were classified as those consuming eggs and dairy ≥1 per month, but red meat, poultry, and fish <1 per month, for at least the previous year. For clear distinction of dietary patterns, the study did not accept pesco-vegetarians (those who consume red meat and poultry <1 per month and fish ≥1 per month) and semi-vegetarians (those who consume red meat, poultry, and fish 1 per month to 1 per week and eggs or dairy at any level) and defined as omnivores those who eat meat, poultry, and fish >1 per week and eggs or dairy at any level (30). For the purpose of this article, selected dietary data will be presented as background characteristics only to help interpret health outcome differences. More detailed dietary analysis will follow in a separate publication. Definitions of terms describing different types of plant-based diets used in this article are presented in **Supplemental Table 1**.

### Outcomes

Our outcomes were anthropometry, body composition, bone health, CVD risk markers, and micronutrient status [iron, B-12, and 25-hydroxy vitamin D (25(OH)D)]. These were measured after dietary data were collected during the child's 1-d visit to the clinic, from September 2014 to July 2017.

#### Anthropometry and body composition

Weight and height; mid-thigh, waist, and hip girths; and biceps, triceps, subscapular, and suprailiac skinfolds were all measured by 2 trained raters according to the standard operating procedures of University College London (UCL) Institute of Child Health. The digital scales (Seca 86l) were regularly calibrated. Height was measured with a portable stadiometer to the nearest 0.5 cm (Seca 213), skinfolds with calipers (Harpenden), and girths with a nonstretchable tape. Body composition was assessed using deuterium (D_2_O) dilution to measure total body water (TBW, liters), using an oral dose equivalent to 0.05 g/kg body weight. Saliva samples were collected using cotton wool swabs at baseline and 4 h after dosing. Isotopic enrichment of saliva samples and the dose administered was determined by isotope ratio mass spectrometry (Gasbench-Delta XP system; ThermoFisher). Lean mass (used synonymously here with fat-free mass) was calculated from TBW using published hydration coefficients (31), and fat mass was calculated as the difference of body mass and lean mass. We normalized body composition for height by dividing by height squared, giving the lean mass index (LMI) and fat mass index (FMI) in the same kg/m^2^ units as BMI. Body composition *z* scores were derived from UK reference data (31).

Total body bone mineral content (BMC) and lumbar spine BMC were assessed by dual-energy X-ray absorptiometry (Lunar Prodigy Advance). For the calibration of the densitometer, a daily quality control procedure was performed. In addition, an anthropometric spine phantom was scanned at least twice weekly. The technician was blind to participants’ dietary exposure. The subject wore light indoor clothing. We extracted BMC for the total body minus the head (TBLH BMC) and the L2–L4 region (L2–L4 BMC), along with the corresponding bone areas, to correct results for bone size. For this purpose, we also calculated bone mineral apparent density (BMAD) using the Carter method, which adjusts BMC for calculated bone volume rather than bone area (32), using data for age, sex, BMC, and bone area for L2–L4. We used UK reference data (33) to obtain BMAD *z* scores.

#### Cardiovascular risk and micronutrient status

Fasting blood (15 mL) was drawn between 08:00 and 10:00. Total cholesterol, HDL cholesterol, LDL cholesterol, VLDL cholesterol, and triglycerides were analyzed by agarose gel electrophoresis (A15 Biochemistry Analyser; Biosystems). The complete blood count was determined by the impedance method (Coulter LH 750). Fasting glucose was analyzed by an enzymatic spectrophotometric method (A15 Biochemistry Analyser). Plasma vitamin B-12 and homocysteine were determined by chemiluminescent microparticle immunoassay using commercial kits (Architech i1000SR Analyzer; Abbott). Insulin was determined by immunoradiometric assay (KIP1251 kit; DiaSource). Insulin-like growth factor 1 (IGF-1) was determined by radioimmunoassay (KIP1589 kit; DiaSource), using the Automatic Gamma counter 1470 Wizard (Perkin Elmer). Insulin growth factor binding protein 3 (IGFBP-3) was determined by the sandwich ELISA method (E03A kit; BioVendor) on an ELISA Plate Reader (PowerWave XS; Bio-TEK). The IGF-1/IGFBP-3 molar ratio was calculated according to the following formula: 1 ng/mL IGF-1  =  0.130 nmol IGF-1 and 1 ng/mL IGFBP-3  =  0.036 nmol IGFBP-3 (34). 25(OH)D was measured by chemiluminescent immunoassay (IDS iSYS Analyser). Ferritin was ascertained by immunochemiluminescence and high-sensitivity C-reactive protein (hs-CRP) by immunoturbidimetry (Cobas 600). hs-CRP and ferritin were analyzed from frozen 3-mL samples remaining 3 y after the original data collection started. Homeostasis model assessment (HOMA-IR) was used to assess insulin resistance, calculated as fasting insulin (μIU/ml) × fasting glucose (nmol/L)/22.5 (35). Nurses and laboratory staff were blinded to dietary exposure. Systolic and diastolic blood pressure were measured using an electronic blood pressure monitor (OMRON 7080) after a 10-min rest, with the child seated and quiet.

Carotid intima-media thickness (cIMT) was evaluated by ultrasonography. All measurements were performed by the same examiner blinded to dietary exposure using a Hitachi Aloka Prosound Alpha 6 and a 5.5- to 12.5-MHz probe. cIMT was measured bilaterally on the common carotid arteries according to methodology described previously (36).

### Ethics

The study was approved by Ethical Committees of UCL and the Children's Memorial Health Institute in Warsaw, Poland, where the study took place. Parents gave written informed consent, and children assented to participate. All participants were offered a nutritional consultation by a clinical dietitian on the day data collection took place. Parents were contacted immediately and given additional nutritional or medical advice if abnormal results were found.

### Statistical analyses

To describe the background characteristics of the diet groups, means and SDs or medians and IQRs were calculated. All dietary background characteristics were expressed as medians, as distributions of nutrient intakes have a right-skewedness. To test the null hypothesis of no difference between the groups, χ^2^, ANOVA or Kruskal–Wallis tests were applied.

For anthropometric outcomes ascertained by 2 raters, we confirmed interrater reliability by computing interclass correlation coefficients and *t* tests of differences between raters’ means. To compare means in the main outcomes across diet groups, we used linear regression models, with vegetarians or vegans compared with the reference group of omnivores. Cluster–robust standard errors were used to calculate 95% CIs to account for clustering of siblings (37). We natural log-transformed outcomes that were not symmetrically distributed (HOMA-IR, VLDL cholesterol, triglycerides, hs-CRP, TBLH BMC, L2–L4 BMC, ferritin, and homocysteine), with differences between groups in these outcomes expressed on a percentage scale (38). This approach was selected because models fitted on the log scale improve the numerical quality of the estimation procedure, whereas CIs for models fitted on the original scale would be large and asymmetric and hence difficult to interpret. However, all estimates and their CIs in the original scale are given in the supplementary material.

We excluded 2 physiologically implausible values (insulin: 29.2 µIU/ml; hs-CRP: 15.79 mg/dL) and divided in 2 the lowest detectable concentration levels of 2 variables, vitamin B-12 and 25(OH)D, that had values <69 pmol/L and <17.5 nmol/L, respectively, to address truncation due to limits of detection of the instrument. The blood pressure monitor failed in those with arm girth <17 cm and >22 cm (*n* = 39); all blood pressure data were therefore excluded from analysis.

Directed acyclic graphs (DAGs) were used to state our assumptions about the interrelations of numerous variables, including background characteristics of dietary groups, associated with the exposure and each set of outcomes and exposure correlates (namely, anthropometry and body composition, bone, CVD risk, iron and vitamin B-12, 25(OH)D, and nutritional intake). This helped us identify a minimum set of confounders to control for (39) according to the most recent theoretical and methodological developments in casual inference (40).

Linear regression models were then fitted for each set of outcomes on diet group that controlled for the relevant (often different) potential confounders. The simplest models included diet group (the exposure) and—if relevant for the outcome—age and sex (model 1). These are presented to aid elucidation of the effect of confounding present in the data. A more complex model (model 2) included further confounders identified by the relevant DAG. Additional models were fitted for some outcomes where mediators (i.e., variables assumed to be on the causal pathway from exposure to outcome) were also controlled for to examine possible pathways of association, assuming that no additional confounders may be at play (model 3). Confounders that had biologically plausible nonlinear relations with the outcome (birth weight, gestational age, maternal prepregnancy BMI) were categorized into fifths and used in the analysis as categorical variables. In the analyses of serum parameters of vitamin B-12 and 25(OH)D, dietary groups were further separated into whether or not the child was given vitamin supplements or vitamin-fortified foods. Seasonality in concentrations of vitamin 25(OH)D was adjusted for by including sine and cosine functions of the day of the year of the blood draw in models with this outcome (41, 42).

Multiple imputation using chained equations (43) was applied to deal with missing values that affected some explanatory variables (birth weight, gestational age, maternal prepregnancy BMI, average CPM, paternal education and height, religion, FMI, LMI), under the assumption of missing at random (44).

Separate to the above, in secondary analyses, ordinal logistic regression analysis was used to compute marginal predictions of the prevalence of several categories of inadequate status of vitamin B-12, iron, and cholesterol in the 3 diet groups. Pairwise comparisons of the marginal predictions were used. The ordinal logistic models included the indicators of diet group and confounders identified by the respective DAGs for the corresponding continuous outcomes. Probable and possible vitamin B-12 deficiency were defined as <148 pmol/L and 148–258 pmol/L, respectively (45). Iron deficiency anemia was defined, following WHO (46), as mild [hemoglobin (HBG) 11.0–11.4 g/dL], moderate (HBG 8.00–10.9 g/dL), or severe (HBG<8 g/dL). Cutoffs for abnormally low serum ferritin concentrations were defined as <15 µg/L, following WHO (47), that identified it as depleted iron stores. Pediatric LDL cholesterol values were classified, following the Expert Panel on Integrated Guidelines for Cardiovascular Health and Risk Reduction in Children and Adolescents (48), as high (≥130 mg/dL), borderline (110–129 mg/dL), or acceptable (<110 mg/dL) and HDL cholesterol as low (<40 mg/dL), borderline (40–45 mg/dL), or acceptable (>45 mg/dL). The results of complete case (CC) and multiple imputation (MI) analyses were compared. All statistical analyses were performed in Stata release 13.1 (StataCorp). A 2-sided *P* value of 0.05 was used as the threshold for statistical significance.

This investigation has an exploratory nature, as some of the health parameters have not been investigated previously in this group, especially in vegans. Hence, corrections for multiple testing were not carried out. Another reason is that this study aimed to assess the safety of PBDs in children, which is more important than detecting differences in their potential CVD benefits, and correction for multiple testing could have obscured evidence suggesting adverse effects. However, the percentage of false-positive results is likely to be lower than that expected from the number of tests in this study, as several health outcomes were tested with more than one method and, in those cases, are affected by a single biological relation.

## Results

### Background characteristics

**FIGURE 1:**
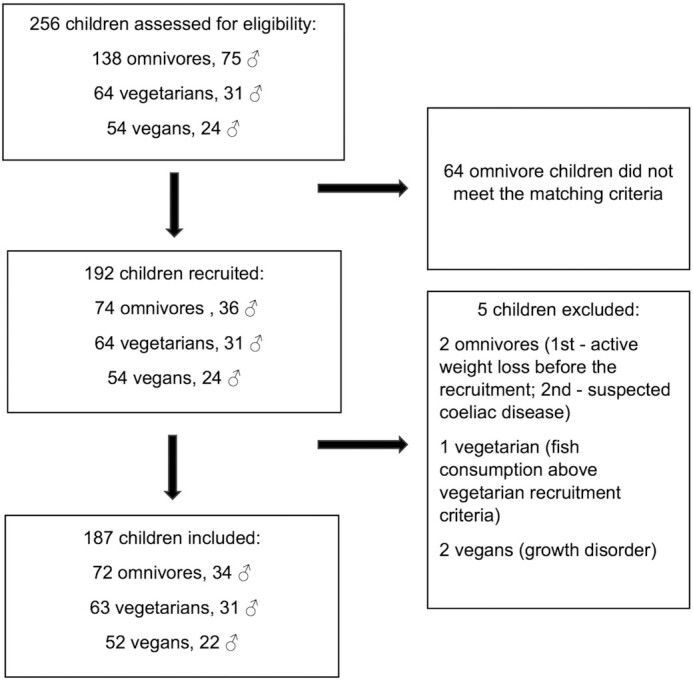
Flow diagram of study from recruitment to inclusion.

We assessed 256 children for eligibility and excluded 64 omnivores who did not meet the matching criteria. We thus recruited 192 children, of whom 74 were omnivores (36 boys), 64 were vegetarians (31 boys), and 54 were vegans (24 boys) (**Figure 1**). Five were disqualified for not fulfilling inclusion criteria. The reasons included suspected celiac disease and recent active weight loss (2 omnivore boys), consuming fish more than once a month (1 girl from the vegetarian group), and suspected growth disorder due to abnormal IGF-1 and growth hormone concentrations (2 vegan boys). This left 187 children in the analysis: 72 omnivores (34 boys), 63 vegetarians (31 boys), and 52 vegans (22 boys). **Table 1** summarizes background characteristics by diet group.

**TABLE 1:** Background characteristics by diet groups^1^

Characteristic	Omnivore	Vegetarian	Vegan	*P* value
Age,^2^ y	7.7 ± 1.7	7.6 ± 1.6	7.6 ± 1.8	0.85^3^
Sex (boys)^4^	34 (47.2)	31 (49.2)	22 (42.3)	0.75^5^
Socioeconomic characteristics				
Residence^4^				
City	55 (76.4)	49 (77.8)	37 (71.2)	0.69^5^
Village	17 (23.6)	14 (22.2)	15 (28.8)	0.69^5^
Maternal smoking^4^	4 (5.6)	8 (12.7)	0 (0.0)	0.02^5^
Paternal smoking^4^	5 (7.0)	5 (7.9)	0 (0.0)	0.13^5^
Maternal education^4^				
Secondary	4 (5.6)	10 (15.9)	10 (19.2)	0.05^5^
Tertiary	68 (94.4)	53 (84.1)	42 (80.8)	0.05^5^
Paternal education^4^				
Secondary	16 (22.2)	20 (33.9)	14 (26.9)	0.33^5^
Tertiary	56 (77.8)	39 (66.1)	38 (73.1)	0.33^5^
Religion^4^				
None	9 (12.5)	37 (59.7)	28 (54.9)	<0.001^5^
Christian	63 (87.5)	22 (35.5)	12 (23.5)	<0.001^5^
Other	0 (0.0)	3 (4.8)	11 (21.6)	<0.001^5^
Perinatal characteristics				
Gestation age,^2^ wk	39.0 ± 1.5	39.2 ± 1.9	38.8 ± 1.9	0.57^3^
Birth weight,^2^ g	3415 ± 455	3355 ± 582	3233 ± 545	0.18^3^
Maternal height,^2^ cm	167.2 ± 6.2	167.1 ± 6.0	168.2 ± 6.4	0.55^3^
Paternal height,^2^ cm	181.0 ± 7.1	180.0 ± 6.1	182.0 ± 7.3	0.27^3^
Breastfeeding,^6^ mo	12.0 (8.0, 16.5)	13.0 (7.0, 18.0)	18.0 (9.0, 24.0)	0.06^7^
Breastfed until 6 mo^4^	61 (84.7)	54 (85.7)	46 (88.5)	0.83^5^
Exclusively breastfed until 6 mo^4^	52 (72.2)	40 (63.5)	37 (71.1)	0.51^5^
Formula introduction timing^4^				
Never formula fed	24 (33.8)	28 (44.4)	31 (60.8)	<0.001^5^
1–5 mo	15 (21.1)	21 (33.3)	12 (23.5)	<0.001^5^
≥6 mo	32 (45.1)	14 (22.2)	8 (15.7)	<0.001^5^
Maternal prepregnancy BMI,^2^ kg/m^2^	22.5 ± 3.4	21.2 ± 2.5	21.9 ± 5.4	0.16^3^
Maternal diet in pregnancy^4^				
Meat eater	64 (97.0)	18 (30.0)	21 (42.0)	<0.001^5^
Vegetarian	1 (1.5)	29 (48.3)	15 (30.0)	<0.001^5^
Vegan	0 (0.0)	2 (3.3)	5 (10.0)	<0.001^5^
Fish eater	1 (1.5)	11 (18.3)	9 (18.0)	<0.001^5^
Family history of disease^4^				
Family history of hypertension	55 (77.5)	36 (61.0)	30 (66.7)	0.12^5^
Family history of type 2 diabetes	22 (32.4)	14 (25.0)	13 (25.0)	0.57^5^
Family history of coronary heart disease	5 (7.7)	16 (27.1)	10 (20.8)	0.02^5^
Physical activity				
Average movement count per minute^2^	8.9 ± 2.4	9.2 ± 2.2	9.8 ± 2.6	0.17^3^
Sedentary activity,^2^ min/d	357.7 ± 81.7	331.8 ± 76.0	335.2 ± 85.6	0.18^3^
Light activity,^2^ min/d	396.4 ± 61.2	403.5 ± 71.5	401.6 ± 67.0	0.84^3^
Moderate activity,^2^ min/d	33.1 ± 16.4	31.7 ± 13.9	35.0 ± 14.7	0.56^3^
Vigorous activity,^2^ min/d	9.0 ± 8.1	18.8 ± 69.7	10.7 ± 7.5	0.40^3^
MVPA of ≥60 min/d^4^	10 (16)	12 (23.5)	11 (24)	0.49^5^
Fortification and supplementation practices				
Vitamin B-12 supplement use^4^	5 (6.9)	22 (34.9)	23 (44.2)	<0.001^5^
Vitamin B-12–fortified products use^4^	17 (23.6)	38 (60.3)	34 (65.4)	<0.001^5^
No vitamin B-12 supplement and no B-12 fortification use^4^	52 (72.2)	17 (27)	15 (29)	<0.001^5^
Vitamin D supplement use^4^	27 (37.5)	21 (33.3)	17 (32.7)	0.82^5^

1 Omnivores, *n* = 72; vegetarians, *n* = 63; vegans, *n* = 52. ANOVA, χ^2^ test, and Kruskal–Wallis test were used to test the null hypothesis of no difference between the groups. MVPA, moderate and vigorous physical activity.

2 Values are means ± SDs.

^3^ANOVA (means).

4 Values are *n* (%).

^5^χ^2^ test (percentages).

6 Values are medians (IQRs).

^7^Kruskal–Wallis test (medians).

There were no meaningful differences in age or sex between groups. Overall, most children from all dietary groups lived in cities or towns and came mainly from highly educated families, although there was a trend among the mothers of vegans and vegetarians to be less educated. Vegans were more likely than the other groups to have never been formula fed and to have nonsmoking parents. However, all families from this study compared favorably to the general Polish population in terms of smoking prevalence and breastfeeding duration (49–51). Vegans and vegetarians were more likely than omnivores to have a family history of coronary heart disease and to have atheist parents. The groups did not differ with regard to the remaining perinatal characteristics and socioeconomic status (SES) or PA, both in terms of average movement count and PA intensity.

Supplementation and fortification practices are presented in Table 1. Nearly a third of children on either vegetarian or vegan diets were not given any B-12 supplements or B-12–fortified foods, and around the same proportion used vitamin D supplements. It is worth mentioning that in Poland, milk is not commonly fortified with vitamin D (or vitamin A). Dietary background characteristics are presented in **Supplemental Table 2**. The diet groups varied in their intake of most nutrients. Omnivores had the highest and vegans the lowest estimated intakes of protein; sucrose; total, saturated, and monounsaturated fat; cholesterol; vitamin B-12; and vitamin D. Vegans had the highest and omnivores had the lowest estimated intake of total carbohydrates, starch, dietary fiber, polyunsaturated fat, folate, carotenoids, vitamin C, magnesium, and iron. Vegetarians had the highest estimated intake of calcium, whereas vegans had markedly the lowest. There were no meaningful differences in estimated energy intake. The mean ± SD duration of exposure to meatless diets was 5.3 ± 2.4 y for vegans and 5.9 ± 2.0 y for vegetarians. Although the inclusion criteria stated that the children recruited to the study had to have followed their respective diets for at least 1 y, in actuality, 85% of the vegetarians and vegans had followed their diets for ≥3 y, whereas the remaining 15% had followed their diets for at least 2 y.

### Health outcomes

Minimally adjusted results (model 1) are presented in the tables to appreciate the extent of confounding present in the data. Unless otherwise specified below, only the multivariable-adjusted, multiple-imputed results for mean differences in outcomes between vegetarians or vegans compared with the reference group of omnivores (models 2, 3) are described in the Results section, as they are meant to represent the causal effects of interest. Complete case analyses (**Supplemental Tables 3–8**) and crude means of all outcomes (**Supplemental Table 9**) are included in the supplementary material.

### Anthropometry and body composition

Mean differences with 95% CIs for anthropometric and body composition outcomes of vegetarians and vegans relative to omnivores are presented in **Table 2**. On average, both vegetarians and vegans were shorter than omnivores (∆–0.32 and –0.57 height *z* score, respectively), which corresponded to ∆–1.9 and –3.15 cm, although the difference in vegetarians was nonsignificant. In comparison to omnivores, both vegetarians and vegans had lower thigh *z* scores, whereas vegans but not vegetarians had lower BMI, FMI, and suprailiac and triceps skinfold along with hip *z* scores. However, there was no evidence of differences in LMI, biceps and subscapular skinfold, or waist circumference between dietary groups.

**TABLE 2:** Crude and adjusted mean differences of vegetarian and vegan children relative to omnivore children in anthropometry and body composition^1^

	Model 1^2^		Model 2^3^	
Outcome	Vegetarian	Vegan	Vegetarian	Vegan
	∆ (95% CI)	∆ (95% CI)	∆ (95% CI)	∆ (95% CI)
Height *z* score	–0.45 (–0.77, –0.12)*	–0.55 (–0.97, –0.12)**	–0.32 (–0.68, 0.03)	–0.57 (–1.02, –0.12)*
BMI *z* score	–0.24 (–0.54, 0.06)	–0.50 (–0.82, –0.17)**	–0.31 (–0.64, 0.02)	–0.53 (–0.95, –0.12)^*^
Lean mass index *z* score	0.02 (–0.28, 0.32)	0.20 (–0.13, 0.53)	–0.07 (–0.41, 0.28)	0.07 (–0.32, 0.47)
Fat mass index *z* score	–0.33 (–0.68, 0.01)	–0.78 (–1.14, –0.42)**	–0.29 (–0.65, 0.07)	–0.72 (–1.12, –0.32)**
Biceps skinfold *z* score	0.03 (–0.21, 0.27)	–0.23 (–0.5, 0.06)	0.04 (–0.28, 0.36)	–0.16 (–0.56, 0.23)
Suprailiac skinfold *z* score	–0.0 (–0.35 0.23)	–0.49 (–0.79, –0.19)**	–0.13 (–0.45, 0.2)	–0.57 (–0.97, –0.18)**
Subscapular skinfold *z* score	0.08 (–0.20, 0.36)	–0.31 (–0.64, 30.03)	0.11 (–0.23, 0.45)	–0.23 (–0.68, 0.22)
Triceps skinfold *z* score	–0.13 (–0.43, 0.17)	–0.56 (–0.87, –0.24)**	–0.11 (–0.48, 0.26)	–0.47 (–0.86, –0.09)*
Waist girth *z* score	–0.24 (–0.52, 0.04)	–0.23 (–0.51, 0.05)	–0.28 (–0.61, 0.05)	–0.30 (–0.67, 0.08)
Hip girth *z* score	–0.20 (–0.53, 0.13)	–0.59 (–0.86, –0.31)**	–0.13 (–0.56, 0.29)	–0.58 (–0.94, –0.21)**
Thigh girth *z* score	–0.37 (–0.65, –0.09)*	–0.61 (–0.90, –0.31)**	–0.37 (–0.69, –0.05)*	–0.58 (–0.97, –0.20)**

1 Ranges of participants available for each outcome by diet group were as follows: omnivores, 67–72; vegetarians, 62–63; and vegans, 45–52. *P < 0.05, **P< 0.01. Linear regression was used to test the null hypothesis of no difference between vegetarian and omnivore as well as vegan and omnivore groups. ∆, difference.

2 Model 1: diet group only.

3 Model 2: diet group, maternal height, paternal height, birth weight (fifths), gestational age (fifths), maternal prepregnancy BMI (fifths), average movement count per hour internal *z* score, breastfeeding duration (<6, 6–12, >12 mo), maternal education, paternal education, and area of residence; multiple imputation was used to account for missing data.

### Bone health, cardiovascular risk, and body iron status

Mean differences in bone, cardiovascular, and body iron status outcomes are presented in **Table 3**. Vegetarians and vegans had 7.3% and 15.2%, respectively, lower TBLH BMC than omnivores. These differences attenuated to the null in vegetarians and were attenuated in vegans to ∆–3.7% after adjusting for presumed mediators (height and weight *z* scores, bone area) (model 3). Therefore, the deficit in bone mass in vegetarians and vegans was mostly explained by the effect of diet on body and bone size but not entirely in vegans. For L2–L4 BMC, the deficits relative to omnivores were detected in vegans only (∆–9.3%). They were attenuated to ∆–5.6% after adjusting for the presumed mediators (model 3). These results were confirmed by another approach (BMAD) correcting for bone size, whereby both BMAD *z* score and percentile were significantly lower for vegans only.

**TABLE 3:** Crude and adjusted mean differences of vegetarian and vegan children relative to omnivore children in bone, cardiovascular, and body iron status outcomes^1^

	Vegetarian	Vegan	Vegetarian	Vegan	Vegetarian	Vegan
Outcome group	∆ (95% CI)	∆ (95% CI)	∆ (95% CI)	∆ (95% CI)	∆ (95% CI)	∆ (95% CI)
Bone status^2^	Model 1^3^		Model 2^4^		Model 3^5^	
TBLH BMC,^6^ %	–7.8 (–13.6, –2.1)**	–16.4 (–24.4, –8.4)**	–7.3 (–14.3, –0. 2)*	–15.2 (–25.4, –4.9)**	11 (–1.6, 3.8)	–3.7 (–7.0, –0.4)*
L2–L4 BMC,^6^ %	–5.3 (–10.5, 0.0)	–10.5 (–17.1, –3.9)**	–4.6 (–10.5, 1.3)	–9.3 (–17.6, –1.1)*	–0.05 (–4.6, 3.7)	–5.6 (–10.6, –0.5)*
BMAD *z* score	–0.086 (–0.408, 0.237)	–0.652 (–1.052, –0.253)**	–0.056 (–0.465, 0.353)	–0.615 (–1.099, –0.132)*	—	—
	–3.3 (–11.5, 4.9)	–12.6 (–21.8, –3.4)**	–2.2 (–12.5, 8.1)	–11.3 (–22.4, –0.2)*	—	—
Cardiovascular risk^7^	Model 1^3^		Model 2^8^		Model 3^9^	
Insulin, µIU/ml	0.23 (–0.56, 1.03)	–0.04 (–0.86, 0.78)	0.20 (–0.84, 1.24)	–0.02 (–1.16, 1.12)	0.56 (–0.39, 1.50)	0.69 (–0.31, 1.70)
Fasting glucose, mg/dL ^6^	3.2 (1.0, 5.5)^**^	2.2 (–0.1, 4.6)	3.1 (0.9, 5.4)**	1.9 (–1.0, 4.8)	3.6 (1.4, 5.8)**	2.7 (–0.3, 5.7)
HOMA-IR,^6^ %	9.1 (–2.4, 20.6)	4.7 (–8.2, 17.5)	8.6 (–6.2, 23.4)	4.5 (–11.7, 20.6)	14.1 (0.8, 27.4)*	14.9 (0.1, 29.7)*
Total cholesterol, mg/dL	–9.3 (–19.2, 0.5)	–33.6 (–42.6, –24.6)**	–11.5 (–22.4, –0.6)*	–35.6 (–48.3, –22.9)**	–10.2 (–21.2, 0.9)	–32.1 (–45.1, –19.0)**
HDL cholesterol, mg/dL	–5.0 (–9.5, –0.5)*	–10.6 (–14.7, –6.4)**	–6.5 (–11.1, –1.8)**	–12.2 (–17.3, –7.1)**	–6.8 (–11.6, –2.0)**	–12.7 (–18.2, –7.1)**
LDL cholesterol, mg/dL	–6.2 (–14.4, 2.0)	–23.4 (–31.0, –15.7)**	–6.9 (–15.6, 1.8)	–24.0 (–35.2, –12.9)**	–5.5 (–14.4, 3.3)	–20.5 (–31.8, –9.2)**
VLDL cholesterol,^6^ %	14 (3.0, 25.0)*	0.0 (–13.0, 14.0)	14.0 (1.0, 28.0)*	2.0 (–15.0,18.0)	16.0 (2.0, 30.0)*	6.0 (–12.0, 23.0)
Triglycerides,^6^ %	18.0 (6.0, 29.0)**	3.0 (–12.0, 17.0)	19.0 (5.0, 33.0)**	6.0 (–12.0, 24.0)	22.0 (7.0, 36.0)**	11.0 (–8.0, 29.0)
hs-CRP,^6^ %	–22.0 (–57.0, 14.0)	–47.0 (–80.0, –15.0)**	–38.0 (–81.0, 5.0)	–81 (–123.0, –39.0)**	–34.0 (–80.0, 11.0)	–72.0 (–118.0, –26.0)**
cIMT, mm	0.000 (–0.010, 0.010)	–0.008 (–0.022, 0.006)	–0.001 (–0.013, 0.011)	–0.009 (–0.024, 0.007)	0.000 (–0.012, 0.013)	–0.007 (–0.021, 0.008)
IGFBP-3, ng/mL	65 (–150, 280)	–105 (–348, 139)	43 (–205, 290)	–144 (–437, 150)	105 (–125, 335)	–50 (–317, 217)
IGF-1, ng/mL	–14 (–45, 16)	–14 (–46, 17)	–10 (–43, 24)	–7 (–47, 34)	6 (–24, 35)	20 (–14, 53)
Molar IGF-1/IGFBP-3 ratio	–0.020,(–0.045, 0.004)	–0.014, (–0.040, 0.011)	–0.016 (–0.044, 0.012)	–0.006 (–0.038, 0.027)	–0.005 (–0.030, 0.020)	0.014 (–0.015, 0.042)
hs-CRP values <1,^6^ %	–5.8 (–36.5, 25.0)	–32.0 (59.6, –4.0)*	–15.4 (–52.2, 21.4)	–55.9 (–90.4, –21.4)**	–10.5 (–48.8, 27.8)	–44.9 (–81.7, –8.0)*
Body iron status^10^	Model 1^3^		Model 2^11^		—	
RBC, M/µL	–0.09 (–0.18, 0.01)	–0.23 (–0.33, –0.12)**	–0.07 (–0.17, 0.02)	–0.23(–0.33, –0.12)**	—	—
HGB, g/dL	–0.24 (–0.50, 0.02)	–0.38 (–0.70, –0.06)*	–0.20 (–0.47, 0.07)	–0.37 (–0.69, –0.05)*	—	—
HTC, %	–83.0 (–160.0, –7.0)*	–105.0 (–203.0, –8.0)*	–72.0 (–150.0, 7.0)	–105.0 (–204.0, –5.0)*	—	—
Ferritin,^6^ %	–19.0 (–37.0, –1.0)*	–28.0 (–48.0, –7.0)**	–14.0 (–32.0, 3.0)	–25.0 (–44.0, –5.0)*	—	—

1 * *P* < 0.05, ** *P* < 0.01. BMAD, bone apparent mineral density; cIMT, carotid intima media thickness; HGB, hemoglobin; hs-CRP, high-sensitivity C-reactive protein; HTC, hematocrit; IGF-1, insulin growth factor 1; IGFBP-3, insulin growth factor binding protein 3; L2–L4, lumbar spine L2–L4 bone mineral content; TBLH BMC, total body less head bone mineral content; ∆, difference.

2 Ranges of participants available for each outcome by diet group were as follows: omnivores, 71–72; vegetarians, 62–63; and vegans, 52 (no missing outcome data).

3 Model 1: diet group, age, sex.

4 Model 2: diet group, age, sex, maternal education, religion, urbanicity.

5 Model 3: diet group, age, sex, maternal education, religion, urbanicity, height *z* score (UK), weight *z* score (UK), bone area.

6 Variable log-transformed; results represent percent difference.

7 Ranges of participants available for each outcome by diet group were as follows: omnivores, 68–71; vegetarians, 60–62; and vegans, 52 (no missing outcome data).

8 Model 2: diet group; age; sex; birthweight quintile; gestational age quintile; maternal prepregnancy BMI quintile; breastfeeding at 6, 6–12, and >12 mo; maternal education; paternal education; religion; urbanicity.

9 Model 3: diet group; age; sex; birthweight quintile; gestational age quintile; maternal prepregnancy BMI quintile; breastfeeding at 6, 6–12, and >12 mo; maternal education; paternal education; religion; urbanicity; height *z* score (UK); fat mass *z* score (DXA); lean mass *z* score (DXA).

10 Omnivores, *n* = 72; vegetarians, *n* = 62; and vegans, *n* = 52.

11 Model 2: diet group, age, sex, maternal education, urbanicity, maternal smoking. Linear regression was used to test the null hypothesis of no difference between vegetarian and omnivore as well as vegan and omnivore groups.

Table 3 also shows that diet was associated with differences in several CVD risk factors. Overall, vegans had on average lower total cholesterol, HDL cholesterol, LDL cholesterol, and hs-CRP than omnivores. Further adjustment for presumed mediators (height, fat and lean mass; model 3) only slightly attenuated the magnitude of the differences, except for HDL cholesterol, in which the difference increased. The differences in hs-CRP remained after excluding 3 outlier values (>1 mg/dL).

Vegetarians, in contrast, had lower average total cholesterol and HDL cholesterol, but the magnitude of the difference in relation to omnivores was smaller than that of the vegans. They also had higher average fasting glucose, VLDL cholesterol, and triglycerides. Model 3 shows strengthened differences between omnivores and vegetarians in glucose, HDL cholesterol, VLDL cholesterol, and triglycerides. In this model, the difference in total cholesterol in vegetarians attenuated to the null, and HOMA-IR became significantly higher. There was no evidence of differences in insulin concentrations, a surrogate marker of atherosclerosis (cIMT), IGFBP-3, IGF-1 concentrations, or molar ratio of IGF-1/IGFBP-3 concentrations or across the 3 diet groups.

Mean differences between diet groups in selected serum indicators of iron status are presented in the last part of Table 3. Vegans had lower concentrations of mean RBCs, hemoglobin, hematocrit, and ferritin. Vegetarians did not differ in any of the iron status indicators from the omnivores.

### Serum indicators of vitamin B-12 and vitamin D status

Differences between diet groups in selected serum indicators of B-12 status [serum B-12, homocysteine, mean corpuscular volume (MCV)], addressing variation in supplementation and fortification practices, are presented in **Table 4**. Vegans had lower mean serum B-12 concentrations than omnivores if they were not given vitamin B-12 supplements or B-12–fortified foods (∆–217.6 pmol/L) or if they were given B-12–fortified foods without B-12 supplementation (∆–139.8 pmol/L). In addition, vegans who were not given B-12 supplements or B-12–fortified foods had higher mean homocysteine and MCV concentrations than omnivores. Vegetarians had lower serum vitamin B-12 (∆–90.9 pmol/L) and higher homocysteine than omnivores if they were not given vitamin B-12 supplements or B-12–fortified foods. There were no differences in serum vitamin B-12, mean homocysteine, or MCV concentrations in vegetarians who were given foods fortified with B-12, as well as vegetarians and vegans who were given B-12 supplements and B-12–fortified foods, in comparison to omnivores. Mean differences between groups in serum 25(OH)D are presented in **Table 5**. Vegetarians and vegans who did not use supplements had lower 25(OH)D concentrations (∆–7.1 and ∆–13.3 nmol/L, respectively) than omnivores. Supplementing vegetarians had higher concentrations than omnivores.

**TABLE 4:** Crude and adjusted mean differences of vegetarian and vegan children relative to omnivore children in serum vitamin B-12, homocysteine, and MCV concentrations addressing variation in vitamin B-12 supplementation and fortification practices^1^

	Vegetarian—no supplementation or fortification	Vegetarian—fortification only	Vegetarian—supplementation and fortification	Vegan—no supplementation or fortification	Vegan—fortification only	Vegan—supplementation and fortification
Outcome	∆ (95% CI)	∆ (95% CI)	∆ (95% CI)	∆ (95% CI)	∆ (95% CI)	∆ (95% CI)
Model 1^2^						
Vitamin B-12, pmol/L	–61.1 (–114.7, –7.6)*	2.1 (–69.6, 73.7)	85.9 (–6.1, 177.9)	–183.8 (–251.9, –115.8)**	–104.0 (–192.0, –16.0)*	66.9 (–36.0, 169.9)
Homocysteine,^3^ %	14.0 (0.0, 27.0)*	–5.0 (–15.0, 4.0)	–12.0 (–25.0, 0.0)	48.0 (25.0, 72.0)**	14.0 (–8.0, 36.0)	–10.0 (–24.0, 3.0)
MCV, fL	–0.28 (–2.16, 1.61)	–0.06 (–2.10, 1.98)	–0.63 (–2.58, 1.33)	4.25 (1.35, 7.15)**	0.84 (–1.64, 3.32)	0.91 (–0.65, 2.46)
Model 2^4^						
Vitamin B-12, pmol/L	–90.9 (–156.7, –25.1)**	–26.4 (–101.5, 48.7)	68.1 (–37.4, 173.6)	–217.6 (–305.7, –129.5)**	–139.8 (–235.3, –44.3)**	43.5 (–59.3, 146.4)
Homocysteine,^3^ %	15.0 (0.0, 30.0)*	–2.0 (–14.0, 9.0)	–11.0 (–25.0, 2.0)	50.0 (27.0, 74.0)**	16.0 (–8.0, 40.0)	–9.0 (–24.0, 6.4)
MCV, fL	–0.28 (–2.33, 1.76)	–0.07 (–2.39, 2.24)	–0.61 (–2.67, 1.46)	4.19 (1.19, 7.18)**	0.97 (–1.63, 3.58)	0.83 (–0.99, 2.64)

1 Omnivores, *n* = 71–72; vegetarians—no supplementation or fortification, *n* = 17; vegetarian—fortification only, *n* = 23; vegetarian—supplementation and fortification, *n* = 22; vegan—no supplementation or fortification, *n* = 15; vegan—fortification only, *n* = 14; vegan—supplementation and fortification, *n* = 23. * *P* < 0.05, ** *P* < 0.01. MCV, mean corpuscular volume; ∆, difference.

2 Model 1: dietary group categorized according to supplementation and fortification status.

3 Variable log-transformed; results represent percent difference.

^4^Model 2: dietary group categorized according to supplementation and fortification status, maternal education, and religion. Linear regression was used to test the null hypothesis of no difference between vegetarian and omnivore as well as vegan and omnivore groups.

**TABLE 5:** Crude and adjusted mean differences of vegetarian and vegan children relative to omnivore children in serum D 25 (OH) concentrations addressing variation in vitamin D supplementation practices^1^

	Vegetarian—no supplementation	Vegetarian—supplementation	Vegan—no supplementation	Vegan—supplementation
Outcome	∆ (95% CI)	∆ (95% CI)	∆ (95% CI)	∆ (95% CI)
Model 1^2^				
Serum D 25 (OH), nmol/L	–7.1 (–13.7, –0.4)*	9.2 (0.7, 17.7)*	–13.2 (–20.2, –6.3)**	–2.5 (–11.5, 6.6)
Model 2^3^				
Serum D 25 (OH), nmol/L	–7.1 (–13.8, –0.3)*	9.2 (0.6, 17.7)*	–13.3 (–20.3, –6.2)**	–2.5 (–11.6, 6.6)

1 Omnivores, *n* = 72; vegetarian—no supplementation, *n* = 40; vegetarian—supplementation, *n* = 20; vegan—no-supplementation, *n* = 35; vegan—supplementation, *n* = 17. * *P* < 0.05, ** *P* < 0.01. D 25 (OH), 25 hydroxy vitamin D; ∆, difference.

2 Model 1: dietary group categorized according to supplementation status, age, sex, and seasonality (sine and cosine function of the day of the year of blood draw).

3 Model 2: dietary group categorized according to supplementation status, age, sex, seasonality (sine and cosine function of the day of the year of blood draw), and maternal education. Linear regression was used to test the null hypothesis of no difference between vegetarian and omnivore as well as vegan and omnivore groups.

### Prevalence of abnormal vitamin B-12, hemoglobin, depleted iron stores, and LDL- and HDL cholesterol status

Estimated prevalence and pairwise comparisons of abnormal vitamin B-12, hemoglobin, depleted iron stores, and LDL and HDL cholesterol status in dietary groups are presented in **Table 6**. For most of these comparisons, the estimated prevalence significantly differed between the vegans and the omnivores. The prevalence of probable vitamin B-12 deficiency was 3% in omnivores, 4% among vegetarians, and 13% in vegans. The prevalence of possible B-12 deficiency was 16%, 19%, and 40% in omnivores, vegetarians, and vegans, respectively. The prevalence of moderate iron deficiency anemia was 0% among omnivores and 2% in both vegetarians and vegans. The prevalence of mild anemia was 0% in omnivores, 7% in vegetarians, and 6% in vegans. There were no children with severe iron deficiency anemia. The prevalence of depleted iron stores (serum ferritin <15 µg/L) was 12.8% in omnivores, 18.3% in vegetarians, and 30.2% in vegans. The prevalence of abnormal pediatric LDL cholesterol status with high (≥130 mg/dL) and borderline high (110–129 mg/dL) LDL cholesterol concentrations was 13% and 17% for omnivores, 6% and 10% for vegetarians, and 0% and 1% for vegans, respectively. The prevalence of low (>45 mg/dL) and borderline (40–45 mg/dL) HDL cholesterol was 7% and 12% for omnivores, 15% and 19% for vegetarians, and 26% and 24% for vegans, respectively.

**TABLE 6:** Estimated prevalence of inadequate vitamin B-12, iron, and cholesterol status^1^

Outcome	Omnivore	Vegetarian	Vegan
Vitamin B-12			
Probable deficiency (<148 pmol/L)	3.2 (0.3, 6.0)	3.8 (0.8, 6.8)	13.0 (2.6, 23.4)*
Possible deficiency (≥148–258 pmol/L)	16.5 (7.5, 25.6)	19.2 (10.2, 28.2)	39.9 (27.8, 52.0)*
Hemoglobin			
Moderate deficiency (8.00–10.9 g/dL)	0	1.9 (–0.3, 4.1)	1.6 (–1.3, 4.5)
Mild deficiency (11.0–11.4 g/dL)	0	6.6 (–0.02, 13.3)	5.6 (1.0,10.2)*
Ferritin			
Depleted iron stores (<15 μg/L)	12.8 (0.05, 20.2)	18.3 (8.5, 28.1)	30.2 (16.2, 44.3)*
LDL cholesterol			
High (≥130 mg/dL)	13.3 (2.2, 24.5)	5.7 (1.1, 10.2)	0.4 (–0.4, 1.2)*
Borderline (110–129 mg/dL)	17.0 (9.2, 24.9)	9.7 (4.1, 15.2)	0.9 (–1.0, 2.7)*
Acceptable (<110 mg/dL)	69.6 (55.2, 84.0)	84.7 (76.4, 92.9)	98.7 (96.1, 101.3)*
HDL cholesterol			
Acceptable (>45 mg/dL)	81.3 (70.7, 91.9)	65.9 (53.9, 78.0)	49.2 (34.3, 64.1)*
Borderline (40–45 mg/dL)	11.8 (5.4, 18.1)	19.3 (12.2, 26.4)	24.4 (16.5, 32.4)*
Low (<40 mg/dL)	6.9 (1.6, 12.1)	14.8 (6.9, 22.8)	26.4 (14.0, 38.7)*

1 Values are expressed as percentages (95% CIs); omnivores, *n* = 72; vegetarians, *n* = 62; and vegans, *n* = 51 (52 for hemoglobin and ferritin). * Pairs of estimated prevalences in vegans versus the reference group of omnivores are significantly different at *P* < 0.05. Pairwise comparisons of marginal predictions following ordinal logistic regression were used to test the null hypothesis of no difference between vegetarian and omnivore as well as vegan and omnivore groups. The following covariates were included in the models: vitamin B-12: maternal education, urbanicity, maternal smoking; hemoglobin and ferritin: maternal education, religion; LDL and HDL cholesterol: birth weight quintile, gestational age quintile, maternal prepregnancy BMI quintile, breastfeeding at 6, 6–12, and >12 mo, maternal education, paternal education, religion, urbanicity.

There were no meaningful differences between the CC and MI analyses.

## Discussion

We recruited 3 groups of children consuming varying amounts of animal-source foods, reflected in contrasting macro- and micronutrient intakes. We found differences in several outcomes in vegetarians and vegans relative to omnivores. Vegan children had more favorable values for several cardiometabolic risk factors and lower fat mass but also decreased stature, decreased BMC, and lower blood micronutrient status. Vegetarians unexpectedly showed a less favorable cardiometabolic risk factor profile; however, other differences were less pronounced. Cardiometabolic risk differences persisted after adjusting for body composition, increasing confidence in our hypothesis that diet itself plays a causal role. Our data indicate that low serum B-12 and 25(OH)D could be rectified by supplementation.

Most previous studies of PBDs in children had a limited sample size and heterogenous dietary classification criteria, examined few health parameters, and lacked adequate controls (11). Studies of vegan children addressed mainly anthropometry and/or lacked a reference group (14, 52, 53). Our results are broadly consistent with previous research but provide more comprehensive data. Most other studies showed that anthropometric measures of children following meatless diets were similar to or below the reference group. It was hypothesized that differences in PA might have contributed to lower fat mass, but we found no such difference. This suggests diet itself is the causal factor (11), given lack of differences in energy intake.

It is well established that B-12 deficiency is an avoidable risk of vegan diets per se and that vegans may also be in particular need of vitamin D supplementation when sunlight exposure is limited. However, evidence comes primarily from adults (54, 55), and our study adds new data for both vegan and vegetarian children, demonstrating inadequate B-12 status in unsupplemented diets, better concentrations in fortified diets, and, in vegans, optimal concentrations when diets incorporate fortification and supplements. Likewise, we show significantly lower values of vitamin D in vegetarians and vegans relative to omnivores that are resolved in those who take supplements. We also provide new data showing lower BMC in vegan children but no difference in vegetarians compared with omnivores, adjusting for body size. Finally, we generated novel data showing lower cholesterol and hs-CRP concentrations in vegans but no differences in IGF-1, IGFBP-3, or cIMT in either PBD group compared with omnivores. Although many of the coefficients for between-group differences are of modest magnitude, upward or downward shifts in population distributions affect how many individuals are in high- or low-risk groups. Among adults, vegetarians and vegans tend to have a better cardiometabolic profile than omnivores and ∼25% lower risk of ischemic heart disease (9). Importantly, atherosclerosis starts in childhood and develops into classical CVD risk factors, which track through to adulthood. These risk factors are affected by diet (9), which itself tracks into adulthood (9). Our finding that vegan diets in children are associated with a better CVD profile might potentially contribute to lowering adulthood CVD. However, we also show that poorly planned PBDs might worsen CVD profile already in childhood, and in adults, such diets are linked to adverse CVD outcomes (56).

Beyond CVD risk, our study addresses knowledge gaps regarding the safety of PBDs in children. Our data suggest that restriction of animal-based foods could prevent children from achieving optimal height or bone mineral status and could lead to selected nutritional deficiencies. The shorter height of children consuming PBDs may have mixed implications for long-term health. Taller height is associated with higher social status, and this association may be causal rather than just an artifact of social correlates (57, 58). Taller adult height is associated with lower risk of NCDs (e.g., diabetes, heart disease) but also with a greater risk of diverse cancers (59). However, whether these height differences will persist into adulthood is unclear.

The findings for BMC are concerning. Maximizing pediatric BMC is recommended (61) to promote peak BMC with the aim of reducing osteoporosis and fracture risk in adulthood. We found that vegans have lower BMC even after accounting for smaller body and bone size. It does not seem optimal to enter adolescence, a phase when bone-specific nutrient needs are higher, with a BMC deficit already established. If such deficits are caused by a diet that persists into adolescence, this might increase the risk of adverse bone outcomes later in life.

The main strength of our study is the detailed assessment of diet and health to identify both risks and benefits of specific PBDs. We recruited adequate numbers to detect a ≥0.55-SD difference in outcomes. The diet groups were matched for age, sex, and SES. We addressed a range of known potential confounders, measuring PA objectively and body composition via 3 independent techniques. Our results are corroborated by the children's nutrient profiles. In vegans, high estimated intakes of fiber, folate, vitamin C, carotenoids, and magnesium and low saturated fat, cholesterol, and sucrose indicate an “unprocessed” type of PBD, which may explain their more favorable CVD risk profile. Conversely, their lower protein, calcium, B-12, and vitamin D intakes may explain their less favorable BMC and serum vitamin concentrations. We speculate that protein quality in vegans might have contributed to the BMC findings (60), but further work is merited. The vegetarians’ nutrient intake suggests a more processed type of PBD, which might explain their worse CVD risk profile. Consistent with adult studies (61), higher intakes of non–haem iron (the less bioavailable form) in vegetarians and vegans were accompanied by lower iron status.

The main limitation of our study was its cross-sectional design. We used convenience sampling of vegans and vegetarians as the only feasible method in this hard-to-reach population. Thus, this study was at risk of selection bias, which should be considered a potential alternative explanation for some of the findings. Other limitations include small levels of missing data and faulty operation of the blood pressure monitor, obliging us to discard these data. In addition, homocysteine is less specific than methylmalonic acid as a second-line test in assessing cobalamin disorders (45). However, it is widely used in similar studies and was chosen to increase comparability of our data. Finally, our findings might not be generalizable to children from nonindustrialized settings, other ethnic groups, or versions of PBDs.

Several unanswered questions remain. Assuming validity of our findings regarding decreased height and BMC in vegans and vegetarians, it is unclear which aspects of PBDs can contribute to these outcomes, at what age, or whether supplementation or dietary change can rectify these problems. We do not know the extent and consequences of long-term cardiometabolic benefits or nutritional risks. Additional research and replication of our findings using longitudinal studies are desirable. Our data relate to ages 5–10 y, but the risks and benefits for children of different ages, especially infants, might vary. We propose that physicians and dietitians educate their patients on both potential benefits and risks of PBDs in children, emphasizing potential effects on stature and bone associated with veganism. Vegan and vegetarian children need guidelines on how to eat healthfully, beyond advice on supplementation. Finally, current debates on PBDs and the position statements of expert organizations should focus even more on customizing the advice to vegans compared with vegetarians and different age groups so that the established benefits of these diets are maximized and the risks minimized in the pediatric population.

## Supplementary Material

nqaa445_Supplemental_File

## Data Availability

Data described in the manuscript, code book, and analytic code will not be made available due to further planned works.

## Notes

This study was supported by a single private donor. This individual had no role in the design and conduct of the study; collection, management, analysis, and interpretation of the data; preparation, review, and approval of the manuscript; or the decision to submit the manuscript for publication. This research benefited from funding awarded to the NIHR Great Ormond Street Hospital Biomedical Research Centre. The UCL Institute of Child Health receives a proportion of funding from the Department of Health's National Institute for Health Research Biomedical Research Centre funding scheme. JGS was supported by a Medical Research Council PhD studentship. Any views expressed are those of the authors and not necessarily those of the funders.

Supplemental Tables 1–9 are available from the “Supplementary data” link in the online posting of the article and from the same link in the online table of contents at https://academic.oup.com/ajcn/.

Abbreviations used: 25(OH)D, 25-hydroxy vitamin D; BMAD, bone mineral apparent density; BMC, bone mineral content; CC, complete case; cIMT, carotid intima-media thickness; CPM, counts per minute; CVD, cardiovascular disease; DAG, directed acyclic graph; FMI, fat mass index; HGB, hemoglobin; hs-CRP, high-sensitivity C-reactive protein; IFGBP-3, insulin growth factor binding protein 3; IGF-1, insulin-like growth factor 1; IGFBP-3, insulin growth factor binding protein 3; LMI, lean mass index; MCV, mean corpuscular volume; MI, multiple imputation; NCD, noncommunicable disease; PA, physical activity; PBD, plant-based diet; SES, socioeconomic status; TBLH BMC, total body less head bone mineral content; TBW, total body water; UCL, University College London.
