# Supplementary material for: Growth, body composition, and cardiovascular and nutritional risk of 5- to 10-y-old children consuming vegetarian, vegan, or omnivore diets
Source: Am J Clin Nutr. 2021 Mar 19;113(6):1565–77. doi: 10.1093/ajcn/nqaa445 (PMC8176147; doi:10.1093/ajcn/nqaa445)
Supplement: nqaa445_Supplemental_File [file nqaa445_supplemental_file.pdf]

On-line Supplementary Material

Growth, body composition, and cardiovascular and nutritional risk of 5-10-year-old children consuming vegetarian, vegan or omnivore diets.

Małgorzata A. Desmond; Jakub G. Sobiecki; Maciej Jaworski; Paweł Płudowski; Jolanta Antoniewicz, Meghan K. Shirley, Simon Eaton, Janusz Książyk, Mario Cortina-Borja; Bianca De Stavola; Mary Fewtrell; Jonathan CK Wells

American Journal of Clinical Nutrition

Supplementary table 1. Definitions of terms describing different types of plant -based diets used in this paper.

**Vegetarian diet**	a diet that excludes meat, fish and products made from these.
**Vegan diet**	a diet that eliminates all products of animal origin, including meat, fish along with dairy and eggs.
**Meatless diet**	a diet excluding meat of all animals, including fish.
**Plant-based diet**	a diet with a large percentage of plant-based foods.

Supplementary table 2. Background characteristics by diet groups - crude medians of observed intakes of nutrients in dietary groups[1]

	Omnivore	Vegetarian	Vegan	*p* value[a]
**Energy (kcal)**	1637 (1460, 1799)	1564.0 (1367,1768)	1614 (1350, 1936)	0.41
**Protein (g)**	57.3 (46.9, 64.5)	45.1 (40.5, 51.5)	42.4 (34.9, 48.4)	<0.001
**Carbohydrates(g)**	216 (197, 243)	243 (212, 275)	266 (225, 310)	<0.001
**Starch (g)**	99.0 (82.1, 111.9)	101.6 (86.8, 119.8)	116.1 (92.4, 138.2)	<0.001
**Sucrose (g)**	51.6 (41.8, 69.9)	46.0 (35.7, 56.1)	38.3 (21.0, 53.0)	<0.001
**Fiber (g)**	14.9 (13.0, 20.2)	24.7 (19.1, 30.2)	31.7 (26.3, 39.0)	<0.001
**Fat (g)**	65.7 (51.7, 78.2)	53.6 (47.6, 62.7)	51.7 (39.2, 67.9)	<0.001
**Saturated fat (g)**	25.3 (20.0, 29.8)	17.7 (14.1, 22.2)	10.6 (7.6, 15.3)	<0.001
**Monounsaturated fat (g)**	25.4 (19.8, 31.2)	19.8 (16.6, 23.0)	19.8 (14.9, 29.0)	<0.001
**Polyunsaturated fat (g)**	9.0 (7.5, 11.2)	11.7 (9.0, 15.0)	17.0 (13.5, 21.6)	<0.001
**Cholesterol (mg)**	246 (196, 325)	147 (112, 193)	5.1 (0.0, 29.3)	<0.001
**Folate (mcg)**	230 (189, 286)	295 (266, 376)	412 (322, 469)	<0.001
**Beta carotene equivalents (mcg)**	2696 (1570, 4998)	3432 (2344, 5310)	5318 (3887, 7928)	<0.001
**Vitamin C (mg)**	105 (76.4, 150.0)	135 (104, 183)	183 (120, 245)	<0.001
**Magnesium (mg)**	223 (182, 260)	301 (238, 357)	402 (344, 489)	<0.001
**Calcium (mg)**	547 (421, 689)	607 (480, 712)	376 (307, 546)	<0.001
**Iron (mg)**	8.6 (7.2, 10.1)	11.5 (9.3, 13.0)	13.1 (11.7, 16.8)	<0.001
**Vitamin B12 (mcg)**	2.7 (2.1, 3.3)	2.4 (1.5, 4.9)	1.2 (0.2, 15.0)	0.34
**Vitamin B12 (mcg) without supplementation**	2.7 (2.1, 3.3)	1.7 (1.2, 2.5)	0.5 (0.1, 1.1)	<0.001
**Vitamin D (mcg)**	2.7 (1.6, 11.5)	1.6 (0.8, 9.8)	1.1 (0.2, 9.4)	<0.001
**Vitamin D (mcg) without supplementation**	2.0 (1.3, 3.0)	1.0 (0.6, 2.0)	0.6 (0.2, 1.8)	<0.001

[1]Values are medians (IQR); omnivores n=72, vegetarians n=63, vegans n=52; [a]Kruskall-Wallis test (medians) was used to test the null hypothesis of no difference between the groups.

Supplementary table 3. Crude and adjusted mean differences of vegetarian and vegan children relative to omnivore children in anthropometry and body composition, complete case analysis[1]

Outcome	Model 1[2]		Model 2[3]	
	Vegetarian	Vegan	Vegetarian	Vegan
	Δ[4] (95% CI)	Δ (95% CI)	Δ (95% CI)	Δ (95% CI)
**Height *z*-score**	-0.48 (-0.87, -0.09) *	-0.64 (-1.12, -0.16) *	-0.40 (-0.81, 0.01)	-0.69 (-1.18, -0.21) **
**BMI *z*-score**	-0.19 (-0.56, 0.17)	-0.45 (-0.82, -0.08) *	-0.30 (-0.67, 0.07)	-0.54 (-0.99, -0.10) *
**Lean mass index *z*-score**	0.041 (-0.306, 0.389)	0.269 (-0.120, 0.659)	-0.090 (-0.526, 0.345)	0.042 (-0.406, 0.491)
**Fat mass index *z*-score**	-0.25 (-0.63, 0.13)	-0.79 (-1.22, -0.36) **	-0.24 (-0.63, 0.16)	-0.73 (-1.17, -0.28) **
**Biceps skinfold *z*-score**	0.15 (-0.13, 0.43)	-0.19 (-0.52, 0.15)	0.19 (-0.18, 0.56)	-0.14 (-0.59, 0.31)
**Suprailiac skinfold *z*-score**	-0.05 (-0.41, 0.30)	-0.36 (-0.69, -0.03) *	-0.15 (-0.52, 0.22)	-0.51 (-0.93, -0.09) *
**Subscapular skinfold *z* -score**	0.11(-0.23, 0.45)	-0.22 (-0.60, 0.16)	0.12 (-0.27, 0.51)	-0.21 (-0.69, 0.27)
**Triceps skinfold *z*-score**	-0.08 (-0.43, 0.27)	-0.56 (-0.91, -0.22) **	-0.08 (-0.51, 0.36)	-0.48 (-0.90, -0.06) *
**Waist girth *z* -score**	-0.18 (-0.51, 0.15)	-0.23 (-0.55, 0.08)	-0.27 (-0.63, 0.09)	-0.38 (-0.79, 0.04)
**Hip girth *z*-score**	-0.18 (-0.58, 0.23)	-0.61 (-0.92, -0.30) **	-0.18 (-0.65, 0.29)	-0.70 (-1.08, -0.33) **
**Thigh girth *z*-score**	-0.37 (-0.70, -0.03) *	-0.58 (-0.91, -0.24) **	-0.42 (-0.79, -0.05) *	-0.62 (-1.04, -0.20) **

* indicates statistical significance at p-value < 0.05; ** indicates statistical significance at p-value < 0.01; [1]ranges of participants available for each outcome by diet group were as follows: omnivores – 57-61, vegetarians – 41-42, vegans – 35-42; [2]Model 1**:** diet group only; [3]Model 2**:** diet group, maternal height, paternal height, birthweight (fifths), gestational age (fifths), maternal pre-pregnancy BMI (fifths), average movement count per hour internal z-score, breastfeeding duration (<6, 6-12, >12 months), maternal education, paternal education, area of residence; multiple imputation was used to account for missing data; [4]difference. Linear regression was used to test the null hypothesis of no difference between vegetarian and omnivore, and vegan and omnivore groups.

Supplementary table 4. Crude and adjusted mean differences of vegetarian and vegan children relative to omnivore children in bone outcomes, complete case analysis[1]

Outcome	Model 1[2]		Model 2[3]		Model 3[4]	
	Vegetarian	Vegan	Vegetarian	Vegan	Vegetarian	Vegan
	Δ[5] (95% CI)	Δ (95% CI)	Δ (95% CI)	Δ (95% CI)	Δ (95% CI)	Δ (95% CI)
**TBLH BMC (%)[6]**	-8.1 (-13.8, -2.4) **	-16.7 (-24.8, -8.6) **	-7.4 (-14.5, -0.4) *	-15.4 (-25.7, -5.0) **	1.0 (-1.7, 3.6)	-3.9 (-7.2, -0.6) *
**L2-4 BMC (%)[6]**	-5.8 (-11.0, -0.6) *	-10.6 (-17.3, -4.0) **	-5.0 (-10.8, 0.9)	-9.3 (-17.6, -1.0) *	-0.8 (-4.9, 3.4)	-5.6 (10.7, -0.5) *
**BMAD *z*-score** (29)	-0.11(-0.43, 0.22)	-0.66 (-1.07, -0.26) **	-0.07 (-0.48, 0.34)	-0.62 (-1.11, -0.13)*	—	. —
**BMAD %ile**	-4.0 (-12.1, 4.2)	-12.7 (-22.0, -3.3) **	-2.7 (-13.0, 7.6)	-11.2 (-22.4, 0.0)	. . —	. . —

* indicates statistical significance at p-value < 0.05; ** indicates statistical significance at p-value < 0.01;[1] ranges of participants available for each outcome by diet group were as follows: omnivores – 71-72, vegetarians – 61-62, vegans 51; [2] Model 1: diet group, age, sex; [3] Model 2: diet group, age, sex, maternal education, religion, urbanicity;[4] Model 3: diet group, age, sex, maternal education, religion, urbanicity, height z-score (UK), weight z-score (UK), bone area; [5] difference; [6] variable log-transformed, results represent percent difference. Linear regression was used to test the null hypothesis of no difference between vegetarian and omnivore, and vegan and omnivore groups. TBLH BMC -total body less head bone mineral content; L2-L4 - lumbar spine L2-L4 bone mineral content; BMAD - bone apparent mineral density.

Supplementary Table 5. Crude and adjusted mean differences of vegetarian and vegan children relative to omnivore children in cardiovascular outcomes, complete case analysis[1]

Outcome	Model 1[2]		Model 2[3]		Model 3[4]	
	Vegetarian	Vegan	Vegetarian	Vegan	Vegetarian	Vegan
	Δ[5] (95% CI)	Δ (95% CI)	Δ (95% CI)	Δ (95% CI)	Δ (95% CI)	Δ (95% CI)
Insulin (μUI/mL)	0.35 (-0.53, 1.23)	-0.04 (-0.93, 0.84)	0.23 (-0.91, 1.38)	-0.18 (-1.42, 1.05)	0.61 (-0.45, 1.67)	0.60 (-0.50, 1.70)
Fasting glucose (mg/dL)	3.9 (1.5, 6.3) **	2.0 (-0.6, 4.6)	3.5 (0.9, 6.0) **	1.4 (-1.8, 4.6)	4.0 (1.5, 6.5) **	2.4 (-1.0, 5.8)
HOMA-IR (%)[6]	12.0 (-1.0, 2.4)	4.0 (-9.0, 18.0)	10.0 (-6.0, 25.0)	2.0 (-16.0, 19.0)	15.0 (1.0, 30.0) *	0.13 (-0.02, 0.29)
Total cholesterol (mg/dL)	-11.0 (-22.0, 0.0) *	-36.0 (-46, -27) **	-13 (-25, -1) *	-38.0 (-52, -24) **	-11.0 (-23, 1)	-34 (-48, -20) **
HDL-cholesterol (mg/dL)	-6.8 (-11.5, -2.1) **	-11.2 (-15.8, -6.6) **	-7.6 (-12.6, -2.5) **	-12.0 (-17.4, -6.7) **	-7.8 (-13.0, -2.6) **	-12.3 (-18.1, -6.5) **
LDL-cholesterol (mg/dL)	-6.4 (-15.4, 2.5)	-25.4 (-33.6, -17.3) **	-7.5 (-16.9, 1.9)	-27.0 (-39.0, -15.1) **	-5.9 (-15.6, 3.7)	-23.4 (-35.1, -11.6) **
VLDL-cholesterol (%)[4]	16.0 (3.0, 28.0) *	0.0 (-15.0, 15.0)	18.0 (3.0, 32.0) *	5.0 (-13.0, 22.0)	20.0 (6.0, 35.0) **	11.0 (-7.0, 28.0)
Triglycerides (%)[6]	20 (7.0, 33.0) **	2.0 (-14.0, 18.0)	23.0 (8.0, 38.0) **	8.0 (-11.0, 27.0)	27 (12.0, 41.0) **	15.0 (-3.0, 33.0)
hsCRP (%)[6]	-32.0 (-66.0, 2.0)	-52.0 (-86.0, -18.0) **	-55.0 (-97.0, -13.0) *	-86.0 (-131.0, -42.0) **	-53.0 (-97.0, -8.0) *	-81.0 (-128.0, -33.0) **
cIMT (mm)	0.002 (-0.009, 0.012)	-0.010 (-0.024, 0.004)	-0.001 (-0.012, 0.010)	-0.011 (-0.026, 0.005)	0.000 (-0.011, 0.012)	-0.008 (-0.023, 0.007)
IGFBP3 (ng/mL)	89 (-149, 327)	-100 (-364, 165)	36 (-238, 311)	-176 (-510, 159)	109 (-146, 365)	-37 (-334, 261)
IGF-1 (ng/mL)	-11 (-43, 22)	-9 (-41, 22)	-12 (-47, 22)	-12 (-55, 31)	3 (-29, 35)	17 (-18, 52)
Molar IGF1:IGFBP3 ratio	-0.019 (-0.045, 0.007)	-0.009 (-0.035, 0.017)	-0.020 (-0.050, 0.010)	-0.010 (-0.043, 0.023)	-0.009 (-0.038, 0.019)	0.011 (-0.018, 0.040)
hsCRP values <1 (%)[6]	-15.0 (-44.0, 14.0)	-36.0 (-64.0, -7.0) *	-0.30 (-0.63, 0.04)	-60.0 (-96.0, -24.0) *	-26.0 (-61.0, 9.0)	-52.0 (-90.0, -15.0) **

* indicates statistical significance at p-value < 0.05; ** indicates statistical significance at p-value < 0.01;[1] ranges of participants available for each outcome by diet group were as follows: omnivores – 60-61, vegetarians – 41-42, vegans - 41-42;[2] Model 1: diet group, age, sex;[3] Model 2: diet group, age, sex, birthweight quintile, gestational age quintile, maternal pre-pregnancy BMI quintile, breastfeeding at 6, 6-12 and over 12 months, maternal education, paternal education, religion, urbanicity;[4] Model 3: diet group, age, sex, birthweight quintile, gestational age quintile, maternal pre-pregnancy BMI quintile, breastfeeding at 6, 6-12 and over 12 months, maternal education, paternal education, religion, urbanicity, height z-score (UK), fat mass z-score (DXA), lean mass z-score (DXA).[5] difference; [6] variable log-transformed, results represent percent difference. Linear regression was used to test the null hypothesis of no difference between vegetarian and omnivore, and vegan and omnivore groups. cIMT-carotid intima media thickness; hs-CRP -high sensitivity C-reactive protein; IGF-1-insulin growth factor 1; IGFBP3- insulin growth factor binding protein 3.

Supplementary table 6. Crude and adjusted mean differences of vegetarian and vegan children relative to omnivore children in selected serum indicators of the iron status, complete case analysis[1]

Outcome	Model 1[2]		Model 2[3]	
	Vegetarian	Vegan	Vegetarian	Vegan
	Δ[4] (95% CI)	Δ (95% CI)	Δ (95% CI)	Δ (95% CI)
**RBC (M/µl)**	-0.087 (-0.182, 0.007)	-0.230 (-0.335, -0.124) **	-0.073 (-0.169, 0.023)	-0.226 (-0.327, -0.125) **
**HGB (g/Dl)**	-0.24 (-0.50, 0.02)	-0.38 (-0.70, -0.06) *	-0.20 (-0.47, 0.07)	-0.37 (-0.69, -0.05) *
**HTC (%)**	-83.0 (-160.0, -7.0) *	-105.0 (-203.0, -8.0) *	-72.0 (-150.0, 7.0)	-105.0 (-204.0, -5.0) *
**Ferritin[5] (%)**	-19.0 (-37.0, -1.0) *	-28.0 (-48.0, -7.0) **	-14.0 (-32.0, 3.0)	-25.0 (-44.0, -5.0) *

* indicates statistical significance at p-value < 0.05; ** indicates statistical significance at p-value < 0.01; [1] omnivores n=72, vegetarians n=62, vegans n=52; [2] Model 1**:** diet group, age, sex;[3] Model 2**:** diet group, age, sex, maternal education, urbanicity, maternal smoking;[4] difference; [5] variable log-transformed, results represent percent difference. Linear regression was used to test the null hypothesis of no difference between vegetarian and omnivore, and vegan and omnivore groups.

Supplementary table 7. Crude and adjusted mean differences of vegetarian and vegan children relative to omnivore children in serum vitamin B12, homocysteine and MCV concentrations addressing variation in vitamin B12 supplementation and fortification practices, complete case analysis1[1]

Outcome	Vegetarian -no supplementation or fortification	Vegetarian – fortification only	Vegetarian – supplementation and fortification	Vegan – no supplementation or fortification	Vegan – fortification only	Vegan – supplementation and fortification
**Model 1[2]**						
	**Δ[3] (95% CI)**	**Δ (95% CI)**	**Δ (95% CI)**	**Δ (95% CI)**	**Δ (95% CI)**	**Δ (95% CI)**
**Vit. B12 (pmol/L)**	-61.1 (-114.7, -7.5) *	-1.4 (-75.3, 72.5)	85.9 (-6.1, 177.9)	-183.8 (-251.8, -115.8) **	-95.5 (-187.6, -3.3) *	66.9 (-36.1, 170.0)
**Homocysteine[4] (%)**	13.7 (0.4, 27.0) *	-3.7 (-12.6, 5.1)	-12.0 (-24.5, 0.5)	48.5 (25.1, 71.9) **	14.8 (-8.5, 38.2)	-10.3 (-23.5, 2.9)
**MCV (fl)**	-0.27 (-2.16, 1.61)	-0.04 (-2.15, 2.06)	-0.63 (-2.58, 1.33)	4.25 (1.35, 7.15) **	1.30 (-1.14, 3.74)	0.91 (-0.65, 2.46)
**Model 2[5]**						
**Vit. B12 (pmol/L)**	-91.0 (-156.0, -25.0) **	- 29.4 (-106.3, 47.5)	68.1 (-37.5, 173.7)	-217.7 (-306.0, -129.4) **	-131.0 (-230.2, -31.9) *	43.4 (-59.4, 146.3)
**Homocysteine[4] (%)**	15.1 (0.1, 30.1) *	-2.4 (-13.6, 8.7)	-11.3 (-24.7, 2.1)	50.2 (26.9, 73.5) **	15.9 (-7.9, 39.7)	-8.9 (-24.3, 6.4)
**MCV (fl)**	-0.29 (-2.33, 1.75)	-0.07 (-2.44, 2.30)	-0.61 (-2.68, 1.45)	4.18 (1.18, 7.18) **	1.42 (-1.14, 3.98)	0.82 (-1.0, 2.63)

* indicates statistical significance at p-value < 0.05; ** indicates statistical significance at p-value < 0.01; [1] omnivores n=71-72, vegetarians - no supplementation or fortification n=17, vegetarian – fortification only n=22, vegetarian – supplementation and fortification n=22, vegan – no supplementation or fortification n=15, vegan – fortification only n=13, vegan – supplementation and fortification n=23; [2] Model 1**:** dietary group categorised according to supplementation and fortification status;[3] difference; [4] variable log-transformed; results represent percent difference;[5] Model 2**:** dietary group categorised according to supplementation and fortification status, maternal education, religion. Linear regression was used to test the null hypothesis of no difference between vegetarian and omnivore, and vegan and omnivore groups.

Supplementary table 8. Estimated prevalence of inadequate vitamin B12, iron and cholesterol status, complete case analysis1[1]

Outcome	Omnivore[2]	Vegetarian	Vegan
**Vitamin B12**			
Probable deficiency (<148 pmol/L)	3.1 (0.3, 6.0)	3.8 (0.8, 6.8)	13.0 (2.6, 23.4)
Possible deficiency (≥148–258 pmol/L)	16.4 (7.4, 25.5)	19.3 (10.3, 28.4)	39.1 (26.9, 51.3)
**Haemoglobin**			
Moderate deficiency (8.00-10.9 g/dl)	0	1.9 (-0.3,4.1)	1.6 (-1.3, 4.5)
Mild deficiency (11.0–11.4 g/dl)	0	6.6 (-0.02, 13.3)	5.6 (1.0,10.2)
**Ferritin**			
Depleted iron stores (< 15 µg/l)	12.8 (0.05, 20.2)	18.3 (8.5, 28.1)	30.2 (16.2, 44.3)
**LDL cholesterol**			
High (≥130 mg/dL)	11.7 (0.5, 22.8)	4.9 (0.4, 9.3)	0.4 (-0.4, 1.3)
Borderline (110–129 mg/dL)	18.1 (9.6, 26.6)	10.0 (3.7, 16.3)	1.1 (-1.2, 3.4)
Acceptable (<110 mg/dL)	70.2 (55.1, 85.3)	85.1 (76.1, 94.1)	98.5 (95.4, 101.6)
**HDL cholesterol**			
Acceptable (>45 mg/dL)	78.5 (66.9, 90.1)	63.5 (50.7, 76.3)	49.3 (33.7, 64.8)
Borderline (40–45 mg/dL)	13.4 (6.7, 20.2)	20.3 (12.7, 28.0)	24.5 (16.2, 32.8)
Low (<40 mg/dL)	8.0 (1.8, 14.2)	16.1 (7.6, 24.7)	26.2 (13.6, 38.9)

[1]Values are expressed as percentages (95%CI); [2] ranges of participants available for each outcome by diet group were as follows n=67-72, vegetarians n=52-62, vegans n=43-52. Pairwise comparisons of marginal predictions following ordinal logistic regression were used to test the null hypothesis of no difference between vegetarian and omnivore, and vegan and omnivore groups. The following covariates were included in the models: vitamin B12 – maternal education, urbanicity, maternal smoking; hemoglobin and ferritin – maternal education, religion; LDL and HDL cholesterol: birthweight quintile, gestational age quintile, maternal pre-pregnancy BMI quintile, breastfeeding at 6, 6-12 and over 12 months, maternal education, paternal education, religion, urbanicity.

Supplementary table 9. Mean and standard deviation of all outcomes by diet groups[1]

Outcome	Omnivore	Vegetarian	Vegan
**Weight z-score** (27)	0.31 (0.92)	-0.07 (0.87)	-0.32 (1.06)
**Height z-score**	0.62 (1.04)	0.18 (0.86)	0.08 (1.20)
**BMI z-score**	-0.03 (0.91)	-0.27 (0.85)	-0.53 (0.80)
**Lean mass index z-score**	-0.34 (0.91)	-0.32 (0.74)	-0.14 (0.82)
**Fat mass index z-score**	-0.26 (0.98)	-0.60 (1.04)	-1.05 (0.91)
**Biceps skinfold z-score**	0.25 (0.76)	0.28 (0.60)	0.02 (0.77)
**Suprailiac skinfold z-score**	-0.22 (0.87)	-0.28 (0.82)	-0.72 (0.71)
**Subscapular skinfold z-score**	-0.14 (0.79)	-0.06 (0.80)	-0.45 (0.90)
**Triceps skinfold z-score**	-0.04 (0.95)	-0.20 (0.81)	-0.60 (0.78)
**Waist girth z -score**	0.56 (0.83)	0.32 (0.83)	0.33 (0.74)
**Hip girth z-score**	-0.11 (0.78)	-0.31 (1.09)	-0.70 (0.67)
**Thigh girth z-score**	-0.28 (0.83)	-0.65 (0.80)	-0.89 (0.73)
**Insulin (μUI/mL)**	6.7 (2.4)	6.8 (2.1)	6.6 (1.9)
**Fasting glucose (mg/dL)**	82.80 (6.0)	85.8 (6.6)	84.8 (6.2)
**HOMA-IR**	1.4 (0.5)	1.5 (0.9)	1.4 (0.5)
**Total cholesterol (mg/dL)**	162.4 (26.4)	152.9 (30)	128.8 (19.9)
**HDL-cholesterol (mg/dL)**	56.5 (12.0)	51.5 (12.6)	45.9 (9.5)
**LDL-cholesterol (mg/dL)**	95.8 (22.8)	89.6 (24.3)	72.5 (17.4)
**VLDL-cholesterol (mg/dL)**	10.0 (3.0)	11.9 (4.4)	10.4 (4.0)
**Triglycerides (mg/dL)**	58.1 (17.0)	71.3 (26.4)	61.6 (23.0)
**hsCRP (mg/dL)**	0.15 (0.42)	0.07 (0.10)	0.06 (0.09)
**cIMT (mm)**	0.36 (0.03)	0.36 (0.02)	0.36 (0.03)
**IGFBP3 (ng/mL)**	3.49 (0.66)	3.52 (0.59)	3.38(0.78)
**IGF-1 (ng/mL)**	210 (86.3)	191.1 (87.6)	194.2 (101.8)
**Molar IGF1:IGFBP3 ratio**	0.22 (0.07)	0.20 (0.07)	0.20 (0.08)
**WBC(K/μl)**	6.6 (1.9)	6.4 (1.5)	5.9 (1.5)
**RBC (M/μl)**	4.7 (0.2)	4.6 (0.3)	4.4 (0.3)
**HGB (g/dL)**	13.1 (0.7)	12.8 (0.8)	12.7 (0.9)
**HTC (%)**	39.3 (1.8)	38.4 (2.4)	38.2 (2.7)
**MCV (fl)**	84.4 (3.6)	84.1 (4.2)	86.3 (3.8)
**Ferritin (ng/ml)**	30.5 (18.2)	24.8 (15.6)	22.7 (11.2)
**Vit. B12[2] (pmol/L)**	335.7 (-)	355.9 (-)	261.2 (-)
**Homocysteine[2] (μmol/L)**	5.5 (-)	5.6 (-)	6.2 (-)
**25(OH)D[3] (nmol/L)**	66.6 (17.5)	65.4 (17.1)	57.1 (17.8)
**TBLH BMC (g)**	663.1 (247.6)	586.5 (180.2)	546.9 (204.5)
**L2-L4 BMC (g)**	16.6 (4.8)	15.3 (3.3)	14.6 (4.1)
**BMAD z-score** (29)	-0.91 (0.94)	-1.00 (0.86)	-1.57 (0.99)

Values are means (SDs) unless otherwise indicated. [2] medians obtained using the Kruskal-Wallis test.[3] adjusted for seasonality by adding residuals from regression of 25(OH)D concentrations on sine and cosine functions of the day of blood draw to the overall mean of 25(OH)D.
